# Risk of Positive Sentinel Lymph Node After Neoadjuvant Systemic Therapy in Clinically Node-Negative Breast Cancer: Implications for Postmastectomy Radiation Therapy and Immediate Breast Reconstruction

**DOI:** 10.1245/s10434-019-07643-x

**Published:** 2019-07-29

**Authors:** S. Samiei, B. N. van Kaathoven, L. Boersma, R. W. Y. Granzier, S. Siesling, S. M. E. Engelen, L. de Munck, S. M. J. van Kuijk, R. R. J. W. van der Hulst, M. B. I. Lobbes, M. L. Smidt, T. J. A. van Nijnatten

**Affiliations:** 1 Department of Surgery, Maastricht University Medical Center+, Maastricht, The Netherlands; 2 Department of Radiology and Nuclear Medicine, Maastricht University Medical Center+, Maastricht, The Netherlands; 3 GROW – School for Oncology and Developmental Biology, Maastricht University Medical Center+, Maastricht, The Netherlands; 4 Department of Radiation Oncology, Maastricht University Medical Center+ (MAASTRO Clinic), Maastricht, The Netherlands; 5 Department of Research, Netherlands Comprehensive Cancer Organization, Utrecht, The Netherlands; 6 Department of Health Technology and Services Research, Technical Medical Center, University of Twente, Enschede, The Netherlands; 7 Department of Clinical Epidemiology and Medical Technology Assessment, Maastricht University Medical Center+, Maastricht, The Netherlands; 8 Department of Plastic, Reconstructive, and Hand Surgery, Maastricht University Medical Center+, Maastricht, The Netherlands

## Abstract

**Background:**

Residual axillary lymph node involvement after neoadjuvant systemic therapy (NST) is the determining factor for postmastectomy radiation therapy (PMRT). Preoperative identification of patients needing PMRT is essential to enable shared decision-making when choosing the optimal timing of breast reconstruction. We determined the risk of positive sentinel lymph node (SLN) after NST in clinically node-negative (cN0) breast cancer.

**Methods:**

All cT1-3N0 patients treated with NST followed by mastectomy and SLNB between 2010 and 2016 were identified from the Netherlands Cancer Registry. Rate of positive SLN for different breast cancer subtypes was determined. Logistic regression analysis was performed to determine correlated clinicopathological variables with positive SLN.

**Results:**

In total 788 patients were included, of whom 25.0% (197/788) had positive SLN. cT1-3N0 ER+HER2+, cT1-3N0 ER−HER2+ , and cT1-2N0 triple-negative patients had the lowest rate of positive SLN: 7.2–11.5%, 0–6.3%, and 2.9–6.2%, respectively. cT1-3N0 ER+HER2− and cT3N0 triple-negative patients had the highest rate of positive SLN: 23.8–41.7% and 30.4%, respectively. Multivariable regression analysis showed that cT2 (odds ratio [OR] 1.93; 95% confidence interval [CI] 1.01–3.96), cT3 (OR 2.56; 95% CI 1.30–5.38), grade 3 (OR 0.44; 95% CI 0.21–0.91), and ER+HER2− subtype (OR 3.94; 95% CI 1.77–8.74) were correlated with positive SLN.

**Conclusions:**

In cT1-3N0 ER+HER2+, cT1-3N0 ER−HER2+, and cT1-2N0 triple-negative patients treated with NST, immediate reconstruction can be considered an acceptable option due to low risk of positive SLN. In cT1-3N0 ER+HER2− and cT3N0 triple-negative patients treated with NST, risks and benefits of immediate reconstruction should be discussed with patients due to the relatively high risk of positive SLN.

Neoadjuvant systemic therapy (NST) has become a more common approach for early-stage breast cancer.^1^ NST targets both systemic and locoregional disease sites and can lead to pathologic down-staging. The increasing use of NST has affected the locoregional treatment decisions, including the surgical management of the breast and axillary lymph nodes, and the indications for postmastectomy radiation therapy (PMRT).^1^^–^3

Previous randomized, clinical trials have reported that PMRT is associated with a lower locoregional recurrence rate (LRR), and improved disease-free survival and overall survival.^4^^–^6 The indication for PMRT is not only dependent on the clinical disease stage and patient characteristics, but also on the final pathological disease stage after NST.^7^,^8^ According to the current guidelines, residual axillary lymph node involvement after NST is the most determining factor for the indication of PMRT independent of other risk factors.^9^,^10^ However, axillary lymph node involvement after NST is difficult to predict.

In parallel, breast reconstruction has become an important aspect of breast cancer treatment. Rates of reconstruction at the time of mastectomy (i.e., immediate breast reconstruction) have increased considerably over the past decades.^11^^–^13 This is important given that immediate breast reconstruction not only benefits the quality of life and reduces the adverse psychosocial consequences, but it is also preferred by most patients.^14^^–^18 If PMRT is indicated in women with immediate breast reconstruction, PMRT can adversely affect the aesthetic outcome of the reconstructed breast and increase the complication risks depending on the type of breast reconstruction.^19^^–^21 Preoperative identification of patients who do need PMRT is essential to enable adequate shared decision-making when choosing the optimal timing of breast reconstruction.

Therefore, the purpose of this study was to determine the overall risk of a positive sentinel lymph node (SLN) after NST in cT1-3N0 breast cancer patients and for the different breast cancer subtypes to support preoperative shared decision-making.

## Methods

This study was approved by the Privacy Review Board of the Netherlands Cancer Registry (NCR), managed by the Netherlands Comprehensive Cancer Organization (IKNL). All cT1-3N0 breast cancer patients who had undergone NST (chemotherapy with or without trastuzumab) with subsequent mastectomy and sentinel lymph node biopsy (SLNB), from January 2010 through December 2016, were identified from the NCR.

On-site trained registrars of NCR collect data from patients’ medical records from all hospitals in the Netherlands. Data were collected on age, tumor characteristics (clinical TNM stage and the pathological TNM stage after NST, tumor histology, tumor grade, and receptor status), and treatment regimens (systemic therapy, breast and axillary surgery, radiation therapy, and immediate breast reconstruction). The axillary nodal status was determined before NST administration by ultrasound. Patients were considered cN0 if ultrasound showed no suspicious lymph nodes or in the case of negative tissue sampling. The pathologic examination of the SLN was performed according to the national guidelines.^22^,^23^ The SLN was sliced on at least three levels with 250-μm spacing and stained with hematoxylin and eosin (H&E). In the case of H&E-negative SLN, immunohistochemistry was performed. The SLN outcome was considered positive in the case of micro- and/or macrometastases.^24^ In the case of isolated tumor cells, the SLN outcome was considered negative.^24^

The ER (Estrogen Receptor) and HER2 (Human Epidermal growth factor Receptor 2) status were determined by immunohistochemistry. Immunohistochemistry testing of ER and HER2 was in accordance with the Dutch national guidelines.^22^, ^23^ The method of scoring for ER status was based on the percentage of tumor cells with nuclear staining. ER was classified as positive if the percentage was ≥ 10%. The scoring of HER2 status was based on the membranous staining of invasive tumor cells. The scoring system is categorized according to coloration in 0 (< 10% tumor cells stain), 1+ (> 10% tumor cells stain with weakly intensity), 2+ (> 10% tumor cells stain with moderate intensity), or 3+ (> 30% tumor cells stain with strong intensity). The coloration scores 0 and 1+ were classified as negative, and the score 3+ was classified as positive. In the case of a 2+ equivocal score, fluorescent in situ hybridization (FISH) was performed and the outcome of FISH overruled.

According to the Dutch national guidelines of 2008 and 2012, the indication for systemic therapy was based on age, tumor size, tumor grade, and receptor status.^22^,^23^ Chemotherapy was recommended for: (1) N0 patients ≤ 35 years (except grade 1 tumors of ≤ 1.0 cm); (2) N0 patients ≥ 35 years with tumors 1.1–2.0 cm and grade 2, or tumors > 2.0 cm; (3) N0 patients with HER2+ tumors ≥ 0.5 cm. These guidelines recommended the following chemotherapy regimens: 6 cycles of TAC (docetaxel, doxorubicin, and cyclophosphamide), or 3 cycles of FEC (fluorouracil, epirubicin, and cyclophosphamide), or 4 cycles of AC (doxorubicin and cyclophosphamide) followed by 12 cycles of paclitaxel or 4 cycles of docetaxel. In addition, HER2+ patients were also treated with trastuzumab for a total of 1 year. No additional HER2-targeted therapy was advised between 2010 and 2016.

Descriptive analyses were performed to evaluate the overall rate of a positive SLN and for the different breast cancer subtypes for cT1-3N0 patients. Patients were stratified into four subtypes with ER-positive(+)HER2+, ER-negative(−)HER2+, ER+HER2−, and triple-negative patients. The PR (Progesterone Receptor) was not included in the determination of the ER+HER2+, ER−HER2+, and ER+HER2− subtypes. Univariable logistic regression analysis was applied to determine the association of patient and/or tumor characteristics with a positive SLN. Multivariable logistic regression analysis was used to adjust for potential confounders. Odds ratios (ORs) with 95% confidence interval (CI) were presented. A two-sided *p* value < 0.05 was considered statistically significant. All statistical analyses were performed using the Statistical Package for the Social Sciences (SPSS, version 25, IBM, Armonk, New York, NY).

## Results

A total of 1914 patients were diagnosed with cT1-3N0 breast cancer between January 2010 and December 2016 in the Netherlands and treated with NST followed by mastectomy and SLNB. Patients were excluded if the SLNB had been performed before NST (*n* = 782). Other exclusion criteria were the unknown date of SLNB or NST (*n* = 259), unknown SLNB outcome (*n* = 33), neoadjuvant endocrine therapy (*n* = 30), unknown breast cancer subtype (*n* = 13), and distant metastases at primary breast cancer diagnosis or within 91 days after surgery (*n* = 9). A total of 788 patients (median age, 48 [range, 18–78] years) were included for final analyses, of whom 106 (13.5%) ER+HER2+, 54 (6.9%) ER−HER2+, 474 (60.1%) ER+HER2−, and 154 (19.5%) triple-negative patients. Immediate breast reconstruction was performed in 378 (48.0%) patients, and PMRT was applied to 288 (36.5%) patients. In patients with immediate breast reconstruction, 150 (39.7%) received PMRT. PMRT was delivered in 156 (79.2%) patients with a positive SLN. A complete overview of all patient, tumor, and treatment characteristics is shown in Table 1.

**Table 1:** Patient, tumor, and treatment characteristics

	All patients	ER+HER2+	ER−HER2+	ER+HER2−	Triple-negative
	*n* = 788	*n* = 106	*n* = 54	*n* = 474	*n* = 154
Age (median; range)	48 [18–78]	47 [18–75]	49 [28–78]	49 [24–75]	47 [24–70]
Clinical tumor stage, no. (%)					
T1	151 (19.2)	28 (26.4)	5 (9.3)	84 (17.7)	34 (22.1)
T2	427 (54.2)	52 (49.1)	32 (59.3)	246 (51.9)	97 (63.0)
T3	210 (26.6)	26 (24.5)	17 (31.5)	144 (30.4)	23 (14.9)
Tumor histology, no. (%)					
Ductal	554 (70.3)	90 (84.9)	44 (81.4)	289 (61.0)	131 (85.1)
Lobular	153 (19.4)	5 (4.7)	3 (5.6)	141 (29.7)	4 (2.6)
Other^a^	81 (10.3)	11 (10.4)	7 (13.0)	44 (9.3)	19 (12.3)
Tumor grade, no. (%)					
1	73 (9.3)	5 (4.7)	2 (3.7)	65 (13.7)	1 (0.6)
2	270 (34.3)	34 (32.1)	16 (29.6)	197 (41.6)	23 (14.9)
3	197 (25.0)	36 (34.0)	20 (37.1)	53 (11.2)	88 (57.1)
Unknown	248 (31.5)	31 (29.2)	16 (29.6)	159 (33.5)	42 (27.7)
Adjuvant radiation therapy, no. (%)	288 (36.5)	27 (25.5)	13 (24.1)	214 (45.1)	34 (22.1)
Immediate breast reconstruction, no. (%)^b^	378 (48.0)	42 (39.6)	23 (42.6)	243 (51.3)	70 (45.5)

*ER* Estrogen Receptor, *HER2* Human Epidermal growth factor Receptor 2, Triple-negative negative for ER, PR, and HER2

^a^Adenocarcinoma not further defined, metaplastic carcinoma, mucinous adenocarcinoma among others

^b^Immediate breast reconstruction data were available from January 2011 until December 2016

Of the included patients, 591 (75.0%) had a negative SLN, 69 (8.8%) had micrometastases, and 128 (16.2%) had macrometastases. The overall rate of a positive SLN for cT1-3N0 patients was 25.0% (197/788). The rate of a positive SLN per subtype was 10.4% (11/106) for the cT1-3N0 ER+HER2+, 3.7% (2/54) for the cT1-3N0 ER−HER2+, 35.9% (170/474) for the cT1-3N0 ER+HER−, and 9.1% (14/154) for the cT1-3N0 triple-negative patients. Table 2 shows the SLN outcome for the different breast cancer subtypes.

**Table 2:** SLN outcome after NST for the different breast cancer subtypes

		SLN negative	SLN positive		
		Total	Micrometastases	Macrometastases	Total
ER+HER2+ *N* = 106	cT1N0	26 (92.8)	1 (3.6)	1 (3.6)	2 (7.2)
	cT2N0	46 (88.5)	2 (3.8)	4 (7.7)	6 (11.5)
	cT3N0	23 (88.5)	0	3 (11.5)	3 (11.5)
ER−HER2+ *N* = 54	cT1N0	5 (100)	0	0	0
	cT2N0	30 (93.7)	2 (6.3)	0	2 (6.3)
	cT3N0	17 (100)	0	0	0
ER+HER2− *N* = 474	cT1N0	64 (76.2)	8 (9.5)	12 (14.3)	20 (23.8)
	cT2N0	156 (63.4)	31 (12.6)	59 (24.0)	90 (36.6)
	cT3N0	84 (58.3)	20 (13.9)	40 (27.8)	60 (41.7)
Triple-negative *N* = 154	cT1N0	33 (97.1)	0	1 (2.9)	1 (2.9)
	cT2N0	91 (93.8)	2 (2.1)	4 (4.1)	6 (6.2)
	cT3N0	16 (69.6)	3 (13.0)	4 (17.4)	7 (30.4)

*SLN* sentinel lymph node, *NST* neoadjuvant systemic therapy, *ER* Estrogen Receptor, *HER2* Human Epidermal growth factor Receptor 2, *Triple-negative* negative for ER, PR, and HER2

In the ER+HER2+ subgroup, 7.2% (2/28) of the cT1N0 patients had a positive SLN, 11.5% of the cT2N0 (6/52), and 11.5% (3/26) of the cT3N0 patients. Of these patients with a positive SLN, 27.3% (3/11) had micrometastases, and 72.7% (8/11) had macrometastases. In these ER+HER2+ patients, 86.8% (92/106) had received neoadjuvant trastuzumab.

In the ER−HER2+ subgroup, the cT1N0 and cT3N0 patients had no positive SLN, and 6.3% (2/32) of the cT2N0 patients had a positive SLN. Both cT2N0 patients with a positive SLN had micrometastases. Neoadjuvant trastuzumab was administered in 92.6% (50/54) of these ER−HER2+ patients.

In the ER+HER2− subgroup, 23.8% (20/84) of the cT1N0 patients had a positive SLN, 36.6% (90/246) of the cT2N0, and 41.7% (60/144) of the cT3N0 patients. Of these patients with a positive SLN, 34.7% (59/170) had micrometastases and 65.3% (111/170) had macrometastases.

In the triple-negative subgroup, the rate of a positive SLN for cT1N0 and cT2N0 patients was respectively 2.9% (1/34) and 6.2% (6/97). This increased up to 30.4% (7/23) for the cT3N0 patients. Micrometastases were found in 35.7% (5/14) of the SLN positive patients and 64.3% (9/14) had macrometastases.

Clinical T1-3N0 ER+HER2+, cT1-3N0 ER−HER2+, and cT1-2N0 triple-negative patients had the lowest rate of a positive SLN: 7.2–11.5%, 0–6.3%, and 2.9–6.2%, respectively. Clinical T1-3N0 ER+HER2− and cT3N0 triple-negative patients had the highest rate of a positive SLN: 23.8–41.7% and 30.4%, respectively (Table 2).

The univariable analysis showed that age ≥ 40 years (OR 1.65, 95% CI 1.07–2.53, *p* = 0.022), cT2 stage (OR 1.77, 95% CI 1.09–2.94, *p* = 0.021), cT3 stage (OR 2.78, 95% CI 1.64–4.72, *p* < 0.001), lobular histology (OR 1.77, 95% CI 1.20–2.62, *p* = 0.004), and ER+HER2− subtype (OR 4.83, 95% CI 2.52–9.27, *p* < 0.001) were associated with higher odds of a positive SLN. Tumor grade 2 (OR 0.57, 95% CI 0.33–0.98, *p* = 0.043) and tumor grade 3 (OR 0.21, 95% CI 0.11–0.40, *p* < 0.001) were associated with lower odds of a positive SLN. After adjustment for confounders, the multivariable analysis showed that cT2 stage (OR 1.93, 95% CI 1.01–3.69, *p* = 0.047), cT3 stage (OR 2.73, 95% CI 1.34–5.54, *p* = 0.006), and ER+HER2− subtype (OR 3.82, 95% CI 1.72–8.84, *p* = 0.001) were correlated with higher odds of a positive SLN. Grade 3 (OR 0.44, 95% CI 0.21–0.91, *p* = 0.026) remained correlated with lower odds of a positive SLN. Table 3 shows the univariable and multivariable logistic regression analysis for the outcome of positive SLN after NST.

**Table 3:** Logistic regression analysis for the outcome of positive SLN after NST

	Univariable analysis			Multivariable analysis		
	OR	95% CI	*p* value	OR	95% CI	*p* value
Age (year)						
≤ 40	1 [reference]			1 [reference]		
> 40	1.65	1.07–2.53	*p* = 0.022	1.13	0.63–2.04	0.668
Clinical tumor stage						
T1	1 [reference]			1 [reference]		
T2	1.77	1.09–2.94	*p* = 0.021	1.93	1.01–3.69	0.047
T3	2.78	1.64–4.72	*p* < 0.001	2.73	1.34–5.54	0.006
Tumor histology						
Ductal	1 [reference]			1 [reference]		
Lobular	1.77	1.20–2.62	*p* = 0.004	0.69	0.39–1.23	0.211
Other	1.49	0.89–2.50	*p* = 0.130	1.51	0.74–3.06	0.254
Tumor grade						
1	1 [reference]			1 [reference]		
2	0.57	0.33–0.98	*p* = 0.043	0.66	0.38–1.16	0.151
3	0.21	0.11–0.40	*p* < 0.001	0.44	0.21–0.91	0.026
Tumor subtype						
ER+HER2+	1 [reference]			1 [reference]		
ER−HER2+	0.33	0.07–1.56	*p* = 0.162	0.20	0.02–1.68	0.138
ER+HER2−	4.83	2.52–9.27	*p* < 0.001	3.82	1.72–8.48	*p* = 0.001
Triple negative	0.86	0.38–1.98	*p* = 0.730	0.96	0.35–2.62	*p* = 0.932

*SLN* sentinel lymph node, *NST* neoadjuvant systemic therapy, *OR* odds ratio, *CI* confidence interval, *ER* Estrogen receptor, *HER2* Human Epidermal growth factor Receptor 2, *Triple-negative* negative for ER, PR, and HER2

## Discussion

In this study, nationwide data were used to report on the risk of a positive SLN in cT1-3N0 breast cancer patients, who had undergone NST followed by mastectomy and SLNB, regarding the need for PMRT and therefore the timing of breast reconstruction (immediate or delayed). We found that cT2 stage, cT3 stage, and ER+HER2− subtype were correlated with higher odds of a positive SLN after NST. Grade 3 was correlated with lower odds of a positive SLN after NST. The lowest risk of a positive SLN was in the ER−HER2+ subtype (3.7%), whereas the highest risk of a positive SLN was in the ER+HER2− subtype (35.7%).

At present, there are no results of randomized trials addressing the role of PMRT following NST. The pooled analysis of National Surgical Breast and Bowel Project (NSABP) B-18 and B-27, and several retrospective studies have addressed the advantages of PMRT after NST.^2^,^3^,^7^,^8^,^25^ Available studies showed that age ≤ 40 years, triple-negative subtype, grade 3 tumors, lymphovascular invasion, and advanced clinical and pathological stage are high-risk features associated with LRR and should require PMRT after NST. Residual axillary lymph node involvement after NST is, in particular, an important prognostic factor for LRR and the most determining factor for the indication of PMRT independent of the risk factors.^9^,^26^,^27^ In our study, independent clinicopathological variables have been assessed that are correlated with a positive SLN after NST. The correlation between cT2 stage, cT3 stage, and ER+HER2− subtype with higher odds of a positive SLN after NST is supported by previous research.^27^^–^29 Both clinical tumor size and tumor subtype are independent predictors of pathologic complete response (pCR). We also showed that patients with larger breast tumors and ER+HER2− subtype have the lowest axillary lymph node response to NST. On the other hand, we demonstrated that tumor grade 3 is an independent predictor correlated with lower odds of a positive SLN after NST. This is in line with previous research given that high-grade tumors are associated with higher rates of axillary pCR.^30^^–^33

The increasing use of NST and the inability to predict the axillary lymph node status after NST have raised questions regarding the patients who do need PMRT and increased the complexity of immediate breast reconstruction planning. Immediate breast reconstruction reduces the number of surgical procedures and has a better aesthetic outcome due to decreased scarring and preservation of the breast skin envelope.^34^^–^37 However, there can be potential reconstruction-related complications with performing an immediate breast reconstruction in patients that unexpectedly require additional PMRT after NST.^38^,^39^ The type of reconstruction (i.e., implant vs. autologous) can affect the complication risks in the setting of radiation therapy. PMRT in the setting of implants can adversely affect the aesthetic outcome and is associated with an increased risk of complications, such as capsular contraction and implant failure.^19^,^40^,^41^ Existing data on autologous reconstruction and PMRT found an increased odds of fat necrosis and volume loss, but acceptable results with regard to complications.^21^,^41^^–^45 In patients who elect to undergo immediate breast reconstruction, the potential need for PMRT should be determined before surgery to diminish the risks of unexpected indication for PMRT and preserve patient satisfaction.

Clinical decision-making regarding the need for PMRT and immediate breast reconstruction requires a multidisciplinary approach and careful patient counseling. The results in this study indicate that cT1-3N0 ER+HER2− and cT3N0 triple-negative patients treated with NST have a high-risk of a positive SLN. For these patients, SLNB as a separate procedure preceding to reconstruction can be considered to provide information about the pathologic axillary lymph nodes prior to definitive surgery. If the SLN is positive, the breast reconstruction can then be delayed, or a tissue expander can be placed to preserve the breast skin envelope.^46^ Data about the axillary lymph nodes prior to a mastectomy can improve the surgical plan for breast reconstruction by incorporating the need for PMRT in surgical decision-making. If PMRT is required, these patients should be well informed throughout the entire decision-making process about the possibility of unfavorable outcomes due to irradiation and contribute to the sequence of breast reconstruction and PMRT.

It has not been fully elucidated whether micrometastases in the axillary lymph nodes after NST should be counted as an indicator for treatment with PMRT. Limited data are available on micrometastases and the LRR rate. In the Mamtani et al.^47^ study, 352 T1–T2 breast cancer patients were included, who had undergone primary mastectomy with isolated tumor cells or micrometastases in the axillary lymph nodes. Of these patients, 95% received adjuvant systemic therapy. The LRR rate without PMRT was 2.8% after 6 years and no LRR occurred among the patients who had received PMRT. There was no significant difference in LRR rate for patients treated with or without PMRT. A limitation is that these results only apply to patients treated with adjuvant rather than neoadjuvant systemic therapy so that the resistance or response to NST is not taken into account. In a study by van Nijnatten et al.,^48^ disease-free survival (DFS) and overall survival (OS) of clinically node-positive patients treated with NST and subsequent axillary lymph node dissection were compared between three groups: axillary pCR (ypN0), isolated tumor cells or micrometastases, and macrometastases. They showed that DFS and OS are not significantly different between patients with axillary pCR and isolated tumor cells or micrometastases, but is significantly lower for patients with macrometastases. In our study, micrometastases were considered as node positive as according to the current guidelines.^24^ Micrometastases were detected in 35.0% (69/197) of the positive SLNs. If only macrometastases were considered as node positive, the positive SLN outcome for all subtypes would be considerably lower. For the cT1-3N0 ER+HER2+ patients, this would even mean that none of the patients had a positive SLN and therefore no indication for PMRT. Further data are needed on whether PMRT is indicated in cN0 patients treated in the neoadjuvant setting with chemoresistant disease.

A strength of this study is the nationwide character of the data including general, academic, and cancer centers, and thus all patients in the Netherlands. Also, the large patient population gave us the opportunity to divide patients into subgroups and therefore the ability to determine the risk of a positive SLN for the different breast cancer subtypes. This study also has certain limitations, such as its retrospective design which made it impossible to retrieve missing data on tumor grade or subtype, date of SLNB or NST, and SLNB outcome. Furthermore, based on the available data it was not possible to determine whether a full course of chemotherapy regimen was completed. Lastly, the lymphovascular invasion was not available in the NCR database and could therefore not be included in the analyses.

## Conclusions

In conclusion, these results showed that in cT1-3N0 ER+HER2+, cT1-3N0 ER−HER2+, and cT1-2N0 triple-negative patients treated with NST, immediate breast reconstruction can be considered an acceptable option due to the low risk of a positive SLN and therefore a decreased likelihood of PMRT. However, in cT1-3N0 ER+HER2− and cT3N0 triple-negative patients treated with NST, the risks and benefits of immediate breast reconstruction should be discussed with these patients due to the relatively high risk of a positive SLN and therefore an increased likelihood of PMRT. In these high-risk patients with a desire for immediate breast reconstruction, an SLNB after NST can be performed before breast surgery to determine the need for PMRT and discuss the potential complications of PMRT thoroughly with the patient in the case of a positive SLN prior to the reconstruction. For both situations, this study provides data for adequate shared decision-making.

## References

[1] Kaufmann M (2006). Recommendations from an international expert panel on the use of neoadjuvant (primary) systemic treatment of operable breast cancer: an update. J Clin Oncol.

[2] Fowble BL, Einck JP, Kim DN (2012). Role of postmastectomy radiation after neoadjuvant chemotherapy in stage II-III breast cancer. Int J Radiat Oncol Biol Phys.

[3] Huang EH, Tucker SL, Strom EA (2004). Postmastectomy radiation improves local–regional control and survival for selected patients with locally advanced breast cancer treated with neoadjuvant chemotherapy and mastectomy. J Clin Oncol.

[4] Overgaard M, Hansen PS, Overgaard J (1997). Postoperative radiotherapy in high-risk premenopausal women with breast cancer who receive adjuvant chemotherapy. Danish Breast Cancer Cooperative Group 82b Trial. N Engl J Med.

[5] Overgaard M, Jensen MB, Overgaard J (1999). Postoperative radiotherapy in high-risk postmenopausal breast-cancer patients given adjuvant tamoxifen: Danish Breast Cancer Cooperative Group DBCG 82c randomised trial. Lancet.

[6] Ragaz J, Jackson SM, Le N (1997). Adjuvant radiotherapy and chemotherapy in node-positive premenopausal women with breast cancer. N Engl J Med.

[7] Buchholz TA, Tucker SL, Masullo L (2002). Predictors of local–regional recurrence after neoadjuvant chemotherapy and mastectomy without radiation. J Clin Oncol.

[8] Mamounas EP, Anderson SJ, Dignam JJ (2012). Predictors of locoregional recurrence after neoadjuvant chemotherapy: results from combined analysis of National Surgical Adjuvant Breast and Bowel Project B-18 and B-27. J Clin Oncol.

[9] Recht A, Comen EA, Fine RE (2016). Postmastectomy radiotherapy: an American Society of Clinical Oncology, American Society for Radiation Oncology, and society of surgical oncology focused guideline update. Pract Radiat Oncol.

[10] CBO Kwaliteitsinstituut voor de Gezondheidszorg. Vereniging van Integrale Kankercentra. Guideline ‘Treatment of breast cancer’ (Richtlijn ‘Behandeling van het Mammacarcinoom’). 2017.

[11] Nabon Breast Cancer Audit (NBCA) jaarrapportage 2017. https://dica.nl/media/1583/DICA_Jaarrapportage_2017_-_Registraties.pdf. Accessed 2 June 2018.

[12] Jeevan R, Mennie JC, Mohanna PN, O’Donoghue JM, Rainsbury RM, Cromwell DA (2016). National trends and regional variation in immediate breast reconstruction rates. Br J Surg.

[13] Lang JE, Summers DE, Cui H (2013). Trends in post-mastectomy reconstruction: a SEER database analysis. J Surg Oncol.

[14] Al-Ghazal SK, Sully L, Fallowfield L, Blamey RW (2000). The psychological impact of immediate rather than delayed breast reconstruction. Eur J Surg Oncol.

[15] Dauplat J, Kwiatkowski F, Rouanet P (2017). Quality of life after mastectomy with or without immediate breast reconstruction. Br J Surg.

[16] Elder EE, Brandberg Y, Bjorklund T (2005). Quality of life and patient satisfaction in breast cancer patients after immediate breast reconstruction: a prospective study. Breast.

[17] Santosa KB, Qi J, Kim HM, Hamill JB, Wilkins EG, Pusic AL (2018). Long-term patient-reported outcomes in postmastectomy breast reconstruction. JAMA Surg.

[18] Atisha D, Alderman AK, Lowery JC, Kuhn LE, Davis J, Wilkins EG (2008). Prospective analysis of long-term psychosocial outcomes in breast reconstruction: two-year postoperative results from the Michigan Breast Reconstruction Outcomes Study. Ann Surg.

[19] Lam TC, Hsieh F, Boyages J (2013). The effects of postmastectomy adjuvant radiotherapy on immediate two-stage prosthetic breast reconstruction: a systematic review. Plast Reconstr Surg.

[20] Magill LJ, Robertson FP, Jell G, Mosahebi A, Keshtgar M (2017). Determining the outcomes of post-mastectomy radiation therapy delivered to the definitive implant in patients undergoing one- and two-stage implant-based breast reconstruction: a systematic review and meta-analysis. J Plast Reconstr Aesthet Surg.

[21] Schaverien MV, Macmillan RD, McCulley SJ (2013). Is immediate autologous breast reconstruction with postoperative radiotherapy good practice? A systematic review of the literature. J Plast Reconstr Aesthet Surg.

[22] CBO Kwaliteitsinstituut voor de Gezondheidszorg. Vereniging van Integrale Kankercentra. Guideline ‘Treatment of breast cancer’ (Richtlijn ‘Behandeling van het Mammacarcinoom’). 2008.

[23] CBO Kwaliteitsinstituut voor de Gezondheidszorg. Vereniging van Integrale Kankercentra. Guideline ‘Treatment of breast cancer’ (Richtlijn ‘Behandeling van het Mammacarcinoom’). 2012.

[24] Giuliano AE, Connolly JL, Edge SB (2017). Breast cancer: major changes in the American Joint Committee on Cancer eighth edition cancer staging manual. CA Cancer J Clin.

[25] Wright JL, Takita C, Reis IM (2013). Predictors of locoregional outcome in patients receiving neoadjuvant therapy and postmastectomy radiation. Cancer.

[26] Buchholz TA, Lehman CD, Harris JR (2008). Statement of the science concerning locoregional treatments after preoperative chemotherapy for breast cancer: a National Cancer Institute conference. J Clin Oncol.

[27] Houssami N, Macaskill P, von Minckwitz G, Marinovich ML, Mamounas E (2012). Meta-analysis of the association of breast cancer subtype and pathologic complete response to neoadjuvant chemotherapy. Eur J Cancer.

[28] Barron AU, Hoskin TL, Day CN, Hwang ES, Kuerer HM, Boughey JC (2018). Association of low nodal positivity rate among patients with ERBB2-positive or triple-negative breast cancer and breast pathologic complete response to neoadjuvant chemotherapy. JAMA Surg.

[29] Boughey JC, McCall LM, Ballman KV (2014). Tumor biology correlates with rates of breast-conserving surgery and pathologic complete response after neoadjuvant chemotherapy for breast cancer: findings from the ACOSOG Z1071 (Alliance) Prospective Multicenter Clinical Trial. Ann Surg.

[30] Mougalian SS, Hernandez M, Lei X (2016). Ten-year outcomes of patients with breast cancer with cytologically confirmed axillary lymph node metastases and pathologic complete response after primary systemic chemotherapy. JAMA Oncol.

[31] Murphy BL, Hoskin TL, Heins CDN, Habermann EB, Boughey JC (2017). Preoperative prediction of node-negative disease after neoadjuvant chemotherapy in patients presenting with node-negative or node-positive breast cancer. Ann Surg Oncol.

[32] Vila J, Mittendorf EA, Farante G (2016). Nomograms for predicting axillary response to neoadjuvant chemotherapy in clinically node-positive patients with breast cancer. Ann Surg Oncol.

[33] Kantor O, Sipsy LM, Yao K, James TA (2018). A predictive model for axillary node pathologic complete response after neoadjuvant chemotherapy for breast cancer. Ann Surg Oncol.

[34] Veronesi P, Ballardini B, De Lorenzi F (2011). Immediate breast reconstruction after mastectomy. Breast.

[35] Didier F, Radice D, Gandini S (2009). Does nipple preservation in mastectomy improve satisfaction with cosmetic results, psychological adjustment, body image and sexuality?. Breast Cancer Res Treat.

[36] Salgarello M, Visconti G, Barone-Adesi L (2010). Nipple-sparing mastectomy with immediate implant reconstruction: cosmetic outcomes and technical refinements. Plast Reconstr Surg.

[37] Toth BA, Lappert P (1991). Modified skin incisions for mastectomy: the need for plastic surgical input in preoperative planning. Plast Reconstr Surg.

[38] Ho AY, Hu ZI, Mehrara BJ, Wilkins EG (2017). Radiotherapy in the setting of breast reconstruction: types, techniques, and timing. Lancet Oncol.

[39] Nelson JA, Disa JJ (2017). Breast reconstruction and radiation therapy: an update. Plast Reconstr Surg.

[40] Jagsi R, Momoh AO, Qi J (2018). Impact of radiotherapy on complications and patient-reported outcomes after breast reconstruction. J Natl Cancer Inst.

[41] El-Sabawi B, Sosin M, Carey JN, Nahabedian MY, Patel KM (2015). Breast reconstruction and adjuvant therapy: a systematic review of surgical outcomes. J Surg Oncol.

[42] Kelley BP, Ahmed R, Kidwell KM, Kozlow JH, Chung KC, Momoh AO (2014). A systematic review of morbidity associated with autologous breast reconstruction before and after exposure to radiotherapy: are current practices ideal?. Ann Surg Oncol.

[43] Rochlin DH, Jeong AR, Goldberg L (2015). Postmastectomy radiation therapy and immediate autologous breast reconstruction: integrating perspectives from surgical oncology, radiation oncology, and plastic and reconstructive surgery. J Surg Oncol.

[44] Mirzabeigi MN, Smartt JM, Nelson JA, Fosnot J, Serletti JM, Wu LC (2013). An assessment of the risks and benefits of immediate autologous breast reconstruction in patients undergoing postmastectomy radiation therapy. Ann Plast Surg.

[45] Garvey PB, Clemens MW, Hoy AE (2014). Muscle-sSparing TRAM flap does not protect breast reconstruction from postmastectomy radiation damage compared with the DIEP Flap. Plast Reconstr Surg.

[46] Kuerer HM, Cordeiro PG, Mutter RW (2017). Optimizing breast cancer adjuvant radiation and integration of breast and reconstructive surgery. Am Soc Clin Oncol Educ Book.

[47] Mamtani A, Patil S, Stempel M, Morrow M (2017). Axillary Micrometastases and isolated tumor cells are not an indication for post-mastectomy radiotherapy in stage 1 and 2 breast cancer. Ann Surg Oncol.

[48] van Nijnatten TJ, Simons JM, Moossdorff M (2017). Prognosis of residual axillary disease after neoadjuvant chemotherapy in clinically node-positive breast cancer patients: isolated tumor cells and micrometastases carry a better prognosis than macrometastases. Breast Cancer Res Treat.

